# Predictors and Extent of Institutional Trust in Government, Banks, the Media and Religious Organisations: Evidence from Cross-Sectional Surveys in Six Asia-Pacific Countries

**DOI:** 10.1371/journal.pone.0164096

**Published:** 2016-10-04

**Authors:** Paul R. Ward, Emma Miller, Alex R. Pearce, Samantha B. Meyer

**Affiliations:** 1 Discipline of Public Health, Flinders University, Adelaide, South Australia, Australia; 2 Department of Knowledge Integration, University of Waterloo, Waterloo, Ontario, Canada; 3 School of Public Health and Health Systems, University of Waterloo, Waterloo, Ontario, Canada; Universidad Veracruzana, MEXICO

## Abstract

**Background:**

Building or maintaining institutional trust is of central importance in democratic societies since negative experiences (potentially leading to mistrust) with government or other institutions may have a much more profound effect than positive experiences (potentially maintaining trust). Healthy democracy relies on more than simply trusting the national government of the time, and is mediated through other symbols of institutional power, such as the legal system, banks, the media and religious organisations. This paper focuses on institutional trust–the level and predictors of trust in some of the major institutions in society, namely politics, the media, banks, the legal system and religious organisations. We present analyses from a consolidated dataset containing data from six countries in the Asia Pacific region–Australia, Hong Kong, Japan, South Korea, Taiwan and Thailand.

**Methods:**

Cross-sectional surveys were undertaken in each country in 2009–10, with an overall sample of 6331. Analyses of differences in overall levels of institutional trust between countries were undertaken using Chi square analyses. Multivariate binomial logistic regression analysis was undertaken to identify socio-demographic predictors of trust in each country.

**Results:**

Religious institutions, banks and the judicial system had the highest overall trust across all countries (70%, 70% and 67% respectively), followed by newspapers and TV (59% and 58%) and then political leaders (43%). The range of levels of higher trust between countries differed from 43% for banks (range 49% in Australia to 92% in Thailand) to 59% for newspapers (28% in Australia to 87% in Japan). Across all countries, except for Australia, trust in political leaders had the lowest scores, particularly in Japan and South Korea (25% in both countries). In Thailand, people expressed the most trust in religious organisations (94%), banks (92%) and in their judicial/legal system (89%). In Hong Kong, people expressed the highest level of trust in their judicial/legal system (89%), followed by religious organisations (75%) and banks (77%). Australian respondents reported the least amount of trust in TV/media (24%) and press/newspapers (28%). South Korea put the least trust in their political leaders (25%), their legal system (43%) and religious organisations (45%). The key predictors of lower trust in institutions across all countries were males, people under 44 years and people unsatisfied with the health and standard of living.

**Conclusion:**

We interpreted our data using Fukuyama’s theory of ‘high/low trust’ societies. The levels of institutional trust in each society did not conform to our hypothesis, with Thailand exhibiting the highest trust (predicted to be medium level), Hong Kong and Japan exhibiting medium trust (predicted to be low and high respectively) and Australia and South Korea exhibiting low trust (predicted to be high and medium respectively). Taiwan was the only country where the actual and predicted trust was the same, namely low trust. Given the fact that these predictors crossed national boundaries and institutional types, further research and policy should focus specifically on improving trust within these groups in order that they can be empowered to play a more central role in democratic vitality.

## Introduction

Trust has been a key sociological concept for many years [1–3], although its definition and conceptualisation remains a point of much debate [4]. Trust has been variously defined within and beyond sociological literatures [5]. On this issue, Govier [6] states that “Trust seems warm and fuzzy, somehow good, perhaps a little Pollyannish” (p.3). A generally accepted definition of trust is ‘the mutual confidence that no party to an exchange will exploit the other’s vulnerability’[7] (p. 1133), with a trustworthy person or institution having both good intentions and reasonable competence [6]. A key cultural motif of late modern democratic society is that trust can no longer be simply taken for granted or expected [8] and distrust (or at least healthy scepticism) becomes the norm [9], indeed a vital ingredient of democracy [10]. Building or maintaining trust is of central importance in democratic societies since negative experiences (potentially leading to mistrust) with government or other institutions may have a much more profound effect than positive experiences (potentially maintaining trust), with some authors suggesting that “trust comes on foot and goes away of horseback” [11] (p. 389).

Levi [12] suggests that “a trusting citizenry and a trustworthy government are the *sine qua non* of contingent consent, democracy may well be a prerequisite of an appropriately trusting citizenry and of trustworthy government and is certainly essential for providing institutional protections to citizens whose expression of scepticism and distrust may be the major engine for an even more democratic state” (p. 96). Obviously, healthy democracy relies on more than simply trusting the national government or political leaders of the time, and is mediated through other symbols of institutional power, such as the legal system, banks, the media and religious organisations. These various institutions differ by country, in terms of their legitimacy, stability, transparency, and independence from government, increasing the need to understand the levels and predictors of institutional trust between countries [13]. Tsfati & Cohen [14] argue that the media provides the conduit whereby people can judge the various institutions, stating “it is impossible to trust democracy unless one perceives that the electorate is well and fairly informed, possessing an accurate picture of the issues at hand” (p. 32). In addition, the courts and a commitment to the rule of law are seen as a fundamental part of democracy [15], thereby making it important to analyse public trust in the legal system.

This paper focuses on institutional trust–the level and predictors of trust in some of the major institutions in society, namely politics, the media, banks, the legal system and religious organisations. Herein we present analyses from a consolidated dataset containing data from six countries in the Asia Pacific region–Australia, Hong Kong, Japan, South Korea, Taiwan and Thailand. We have previously published comparative analyses from this dataset on social quality [16], access to healthcare services [17] and interpersonal trust [18] across these countries. There have been a number of studies examining trust in one or more of these institutions within individual countries, although fewer studies undertaking a comparative analyses across countries [13]. Indeed, it has been argued that the majority of research on institutional trust has been undertaken in the US [19–22] or the EU [23–26], which is not directly transferable to other countries with different social, cultural, political and economic systems, thereby creating a need for research across Asian countries [13]. Some studies have utilised the AsiaBarometer survey or the World Values Survey to examine trust in the media across large numbers of countries [13,27] or undertaken primary data collection to compare trust in national governments between Japan, South Korea and Taiwan [28] or between South Korea and the Netherlands [29]. There have also been studies examining trust and social cohesion in single countries in Asia [30,31].

Whilst we focus specifically on institutional trust within this paper, we recognize the dualistic and intertwined nature of interpersonal and institutional trust–the ways in which trust in an individual can impact trust in the institutional they represent and vice versa. The surveys on which our paper is based asked respondents to rate their level of trust in various institutions in their country, but we cannot fully disentangle their trust in individuals, or the contingencies upon which their trust is based, who work for or in some way represent those institutions. The ‘meeting place’ of interpersonal and institutional trust is what Giddens [32] called ‘access points’–the bank teller for the banking system, the doctor for the medical system, the news reporter for the media, the solicitor for the legal system and so on. Govier [6] encapsulates the complexities of studying *either* institutional *or* interpersonal trust when she talked about ‘trust in objects’, saying, “When we assume that an object will serve its function, we are, in effect, assuming that the various people who manufactured and marketed it did their jobs honestly and properly. If these objects do not perform, someone somewhere made a mistake” (p. 16). In this way, for people to trust the media, the newspaper (as the object) needs to be inscribed with trustworthy individuals and institutions, including the company who owns it, the editors who manage it, the journalists who write stories, the scientists who undertake the research that might be written about and so on. Giddens [32] made a particular point about the links between interpersonal and institutional trust, “Although everyone is aware that the real repository of trust is in the abstract system, rather than the individuals who in specific contexts ‘represent’ it, access points carry a reminder that it is the flesh and blood people (who are potentially fallible) who are its operators” (p.85). It is important too to acknowledge that when one does not trust, it cannot be assumed that they therefore distrust, as trust is not a binary concept [33] and distrust and trust are considered to be conceptually and semantically distinct. One’s trust may be considered to fall somewhere on a spectrum between complete trust to complete distrust [34], with distrust suggested in Barbalet (p.373) to be “an indifference to trust relations” [35]. Within the present analysis, we therefore investigate ‘high’ or ‘low’ trust, and rather than ‘trust’ and ‘distrust’.

Hudson [26] argues, following Luhmann [3,36,37] and Hardin [38], that institutional trust is conceptually different to interpersonal trust–the latter being a ‘rational’ consideration once information is gathered in order to make the choice to trust or not [26]. Hudson suggests that in deciding whether or not to trust a national government, an individual “is not considering whether or not they can trust the national government to carry out a political act for them over which they have a choice. Instead they are considering the extent they trust the institution to fulfil its role in a satisfactory manner” (p. 46). In this way, institutional trust considers issues such as whether a newspaper can be ‘trusted’ to present the news accurately and without bias, whether a bank can be ‘trusted’ to keep their money and give it back through an ATM when requested, and whether a political party can be ‘trusted’ to keep their election promises. This concurs with Mishler and Rose’s definition of institutional trust as ‘the expected utility of institutions performing satisfactorily’ [39]. However, this may depend on the individual having some experience with the institution in question, otherwise as Hardin suggests, individuals may not be able to say ‘one way or the other whether they [institutions] are trustworthy’ [40]. Giddens attempts to deal with this issue by suggesting that there must be some social or cultural norms underpinning the decision to trust (outside of actual experience), often based on a constructed characterisation or stylised notion of the institution [6]. Indeed, Fukuyama [41] argues that “trust arises when a community shares a set of moral values in such a way as to create expectations of regular and honest behaviour” (p.153). In the current paper, we only asked questions about institutions that we assumed people would have experience of, and could therefore make a judgement about their extent of trust in them, although they may be making judgements based on media representations and/or shared cultural values of the institutions.

Braithwaite [42] distinguishes two different types of ‘trust norms’ for institutional trust–‘exchange trust norms’ which are built on expectations of predictability, consistency, cautious decision-making, high performance standards–reflective of banks and legal system. The second set of norms are ‘communal trust norms’ which include additional expectations of flexibility, adaptability, helpfulness–more akin to charitable organisations, NGOs, welfare organisations. The institutions used within the current paper most comfortably fit within the ‘exchange trust norms’.

### Conceptual terrain for understanding institutional trust

Fukuyama developed a seminal theory about ‘low trust societies’ and ‘high trust societies’, which we have previously explored in order to understand interpersonal trust (a more expanded overview of his theory can be found there [18]).

As outlined in our previous paper, Fukuyama argues that within low trust societies, social relations/connections are primarily within the family–familial piety–and that these societies will be less trusting of institutions outside of the family [41], “communities with the strongest internal ties will have the weakest bonds with the outside” (p.154). In an Asian context, he provides extensive evidence of the ways in which the family is still the central unit in Confucianist societies and of the impact this has on reduced trust in institutions outside the family. He argues that businesses have developed as family owned/run organisations in Confucianist countries like Taiwan and Hong Kong, but less so in countries like Japan–with South Korea and Thailand having parts of both Confucianist philosophy but also a more open attitude to trusting others due to State attempts allow more foreign investment. In a Confucianist context, Fukuyama argues that “the lack of trust outside the family makes it hard for unrelated people to form groups or organizations” (p.73). He also argues that Japan is a high trust society, alongside countries like the US, the UK and Germany. Whilst he does not explicitly mention trust in Australia, we would suggest that it is closer to the US and UK, and thus may be hypothesised as a high trust society. There have been theoretical [43,44] and empirical [45–47] critiques of Fukuyama’s work on trust (see also [18]). For example, Khodyakov (2007) argues that Fukuyama’s distinction between low- and high-trust societies is unidimensional and therefore does not accurately characterize social trust. However, his critiques are rooted in his analysis of the differences between interpersonal and institutional trust which we agree calls for greater research focus on the multidimensionality of trust. However, this does not impact the use of Fukuyama’s theory in the present research. Empirical critiqueshave tended to examine the levels and predictors of trust between two countries (see for example [45,47]. However, these studies only compared Japan and America, both of which according to Fukuyama are high trust societies and are based in interpersonal rather than institutional trust.

Fukuyama’s theory sets up a key hypothesis for this paper–to test if there is higher institutional trust in non-Confucianist societies as compared to Confucianist societies. Our previous work investigating interpersonal trust within and between these countries extended Fukuyama’s work by identifying high- and low-, as well as medium-trust societies, in addition to high and low trusting population subgroups [18], therefore both corroborating and refuting his theory. Our extension of this theory is applied in the present analysis. In this way, our paper operationalizes and further extends Fukuyama’s sociology of trust.

## Methods and Dataset

### Ethics statement

Appropriate research ethics approvals were obtained within each country to undertake the individual surveys. The authors were granted ethics approval from Flinders University Social and Behavioural Research Ethics Committee to obtain and use the collected data for secondary analysis (project number 5221).

### Data Collection

The data presented in this paper come from a larger survey across six Asia-Pacific counties: Australia (Flinders University), Hong Kong (Chinese University Hong Kong), Japan (Chiba University), South Korea (Seoul National University), Taiwan (National Taiwan University), Thailand (King Prajadhipok Institute). The details and critique of the survey methods across the six countries, and the means by which we met recognised standards for cross-country survey research have been published elsewhere [16–18]. A brief synopsis is provided here for readers to understand the results presented in the paper. Method used within the study countries are also published elsewhere [30,31,48–50].

Various methodological issues related to cross-country research are acknowledged to potentially lead to difficulties in data interpretation. Consequently, we undertook a number of strategies to make each country-specific survey as comparable as possible. We used questions from pre-validated questionnaires, including the World Values Survey [51] and the General Social Survey [52], to develop the questionnaire. The English version of the questionnaire was then validated [53] and subsequently translated into the language of the host country. Consultations were then undertaken with academics from each of the collaborating universities in order to further refine the questionnaires. Pilot studies were then undertaken in each country to validate the questionnaires and to make ensure respondents understood the questions and response categories, reducing the potential for systematic bias. Within the context of this paper, the questions relating to trust in each of the six different institutions were tested for validity in each country. During the pilot studies in each country, participants were asked to explain how they interpreted each of the institutions (e.g. newspapers, media, banks) and the researchers in each country were confident of the validity of each question used within this paper. However, other than for the English version of the questionnaire (used in the Australian survey), no statistical analysis of validity was undertaken of the different language versions of the questionnaire, which is a potential limitation of the results undertaken in this paper.

Data were collected in each country between 2009 and 2010, using either face-to-face or postal survey techniques. The total sample size was 6331: 681 in Hong Kong; approximately 1000 in Australia and Japan; and 1200 in each of South Korea, Taiwan and Thailand. Given these data are now 6–7 years old, it is possible that institutional trust in these countries may have changed due to particular events, particularly when recognizing that trust is a process [43]. For example, the 2011 nuclear disaster at Fukushima in Japan, as a result of a tsunami, may well have impacted trust in political leaders. Similarly, the 2014 coup d’état in Thailand may have impact trust in political leaders, in addition to the legal system. Therefore, care needs to be taken when interpreting the findings from this study in terms of their complete reliability in 2016 or beyond.

This paper presents an analysis of questions on institutional trust. The question appeared in the questionnaire as: How much do you **trust** various **institutions**? (emphasis in original). There were four response categories: Trust them completely; Trust them a little; Do not trust them very much; Do not trust them at all. The survey provided a list of six different groups against which respondents were asked to rate their level of trust: political leaders, press/newspapers, TV/media, judicial/legal system, banks, and religious organisations. The questionnaire also included 11 questions on socio-demographics such as income, gender and age. However, the Japanese survey did not include questions on trust in banks or trust in religious organisations (for cultural reasons determined by the Japanese researchers).

### Data Analysis

After the surveys had been undertaken, merging and cleaning of the dataset was conducted by academics at Seoul National University. Data were subsequently weighted for each country on the basis of age and sex to account for surveys generally under-representing males and younger respondents.

Responses to survey items enquiring about individual’s trust in various groups of people were dichotomised to achieve suitable levels for analysis and a basis for comparison because the original four categories did not yield enough cases in each category to allow multivariate analysis in each country. For all countries, ‘trust them completely’ and ‘trust them a little’ were combined to form a categorical variable labelled ‘higher trust’, whilst responses in the form of ‘do not trust them very much’ and ‘do not trust them at all’ were combined into ‘lower trust’. The dichotomised variable does not have the fine grained differences in trust as the original variable, and this may represent a potential limitation in interpreting the results of the paper. In order to identify whether (socio) demographic attributes of respondents hold predictive qualities for trust invested in different institutions, a pool of categorical predictor variables was generated comprising respondents’ sex, age group, marital status, work status, household income, as well as subjective satisfaction with health, subjective satisfaction with standard of living, and whether respondents reported a chronic health condition.

Chi-Square analyses were conducted to examine the relationship between country and trust in institutions. All chi-square analyses revealed a significant association between different countries and level of trust placed in the different institutions (all chi-square analyses significant at p < .001).

Data were analysed using Stata (Release 13, Stata Corporation, College Station, TX, USA). Binomial logistic regression models were used to investigate associations for all six countries [54]. Due to differences in data collection methods, and changes to survey questions to make them culturally relevant, a few of the independent variables were not available from some countries thus reducing the number of association tests performed. Goodness-of-fit for all models were checked [54,55]. The regression models presented in the paper give details of the statistically significant predictors of institutional trust within each country. We could not perform statistical tests allowing a comparison across the countries (mixed effects models) because of some of the slightly different predictor variables and sampling techniques. However, within the paper we provide tentative comparisons of the regression models across the countries in order to highlight possible similarities and differences requiring further research. Nevertheless, all comparisons made within the paper need to be interpreted with caution.

All demographic predictor variables were entered into the analysis as categorical variables. Binomial logistic regression analyses were performed to examine the independent effects of demographic variables on trust in institutions., For the purposes of the present investigation the method of choice for conducting regression analyses was to enter relevant predictor variables in one block rather than stepwise procedures [56]. As with our previous analysis of interpersonal trust [18] predictor variables that were entered into the model but returned as not significant were in turn tested against models containing only significant predictor variables. This process allowed for the comparison of several models, resulting in a final model containing only variables, which significantly contributed to the model fit. For each outcome variable, predictor variables included in the regression model were checked for multicollinearity. Given the relatively high number of regression analyses presented in this paper, there is a possibility of some spurious regression models, and therefore caution needs to be taken with p values close to 0.05.

## Results

Table 1 presents data on higher trust in different institutions across the six countries. In terms of overall levels of institutional trust across all six institutions, Thailand had the highest level of trust (84%), followed by Hong Kong (69%) and Japan (65%). Conversely, Australia had the lowest level of trust (43%), followed by South Korea (49%) and Taiwan (55%).

**Table 1:** Proportion of respondents indicating higher trust (trust ‘a little’ or ‘a lot’) in institutions.

	n (%)						
	Political Leaders	Press/ Newspapers	TV/ Media	Legal System	Banks	Religious Organisations	Mean %
**Australia**	395 (41)	279 (28)	245 (24)	553 (56)	491 (49)	561 (62)	43
**Hong Kong**	360 (55)	372 (55)	394 (59)	597 (89)	525 (77)	493 (75)	68
**Japan**	247 (25)	871 (87)	740 (74)	733 (74)	*	*	65
**South Korea**	229 (25)	590 (60)	613 (62)	411 (43)	579 (60)	428 (45)	49
**Taiwan**	423 (38)	516 (45)	539 (46)	637 (56)	832 (73)	798 (70)	55
**Thailand**	837 (71)	896 (76)	948 (80)	1038 (89)	1059 (92)	1060 (94)	84
**TOTAL**	2491 (43)	3524 (59)	3479 (58)	3969 (67)	3486 (70)	3340 (70)	61

* These questions were not asked in the survey in Japan

In Thailand, people expressed the most trust in religious organisations (94%), banks (92%) and in their judicial/legal system (89%). In Hong Kong, people expressed the highest level of trust in their judicial/legal system (89%), followed by religious organisations (75%) and banks (77%). Australian respondents reported the least amount of trust in TV/media (24%) and press/newspapers (28%). South Korea put the least trust in their political leaders (25%), their legal system (43%) and religious organisations (45%).

In terms of trust in the individual institutions, religious institutions, banks and the judicial system had the highest overall trust across all countries (70%, 70% and 67% respectively), followed by newspapers and TV (59% and 58%) and then political leaders (43%). The range of levels of higher trust between countries differed from 43% for banks (range 49% in Australia to 92% in Thailand) to 59% for newspapers (28% in Australia to 87% in Japan). Across all countries, except for Australia, trust in political leaders had the lowest scores, particularly in Japan and South Korea (25% in both countries).

Table 2 shows the statistically significant differences (assessed by Chi square) between higher/lower trust in each of the countries for each of the institutions. In terms of the size of the differences in trust between countries, we calculated the Risk Ratios (RR) of ‘lower trust’ in each of the institutions, using Thailand as the reference category because it had the highest levels of trust in most institutions. Respondents in Hong Kong, Japan, South Korea, Taiwan and Australia were significantly more likely to have lower trust in political leaders than Thai respondents, with RRs ranging from 1.6 (95% CI 1.4–1.8) in Hong Kong to 2.6 in Japan and South Korea (95% CI 2.4–2.9 in both countries). Australians expressed almost 3 times lower trust than Thais in press/newspapers (RR3; 95% CI 2.7–3.3) and 4 times lower trust in TV (RR3.8; 95% CI 3.4–4.3) and the judicial system (RR4; 95% CI 3.3–4.7). South Koreans were approximately five times less likely to trust their legal system (RR5.1; 95% CI 4.3–6.0) and banks (RR 4.9; 95% CI 4.0–6.0) than respondents in Thailand. Australian respondents demonstrated the lowest comparative levels of trust in banks, being over 6 times lower trusting than Thais (RR6.2; 95% CI 5.1–7.6).

**Table 2:** Differences in ‘higher trust’ in institutions between countries.

	Chi-square	RR (95% CI)					
		THD	HK	JPN	TWN	South K	AUS
**Political Leaders**	*x*^2^ (5, 5807) = 688.16, p<0.001	ref	1.58 (1.40–1.79) p<0.001	2.63 (2.38–2.89) p<0.001	2.16 (1.94–2.39) p<0.001	2.62 (2.37–2.89) p<0.001	2.05 (1.85–2.28) p<0.001
**Press/ Newspaper**	*x*^2^ (5, 5982) = 958.78, p<0.001	ref	1.84 (1.61–2.09) p<0.001	0.53 (0.44–0.64) p<0.001	2.28 (2.04–2.55) p<0.001	1.63 (1.43–1.85) p<0.001	2.95 (2.65–3.29) p<0.001
**TV/Media**	*x*^2^ (5, 6004) = 882.06, p<0.001	ref	2.09 (1.81–2.42) p<0.001	1.32 (1.13–1.54) p<0.001	2.72 (2.40–3.09) p<0.001	1.91 (1.66–2.20) p<0.001	3.83 (3.40–4.32) p<0.001
**Legal System**	*x*^2^ (5, 5918) = 781.99, p<0.001	ref	0.97 (0.74–1.27) p = 0.838	2.37 (1.96–2.87) p<0.001	3.92 (3.29–4.67) p<0.001	5.07 (4.28–6.01) p<0.001	3.95 (3.32–4.71) p<0.001
**Banks**	*x*^2^ (4, 4957) = 552.63, p<0.001	ref	2.76 (1.81–2.42) p<0.001	*	3.32 (2.68–4.12) p<0.001	4.90 (3.98–6.02) p<0.001	6.24 (5.10–7.63) p<0.001
**Religious orgs**	*x*^2^ (4, 4777) = 612.19, p<0.001	ref	2.19 (1.96–2.45) p<0.001	*	1.92 (1.79–2.06) p<0.001	3.05 (2.80–3.32) p<0.001	2.39 (2.21–2.59) p<0.001

* These questions were not asked in the survey in Japan

Tables 3 to 8 provide details of the multivariate regression models for lower trust in each of the institutions in each country where a regression model could be calculated.

**Table 3:** Multivariate regression models for lower trust (trust ‘not at all’ or ‘not much’) in Political Leaders in each country.

Country	Characteristic	Risk Ratio (95% CI)	Risk Difference (95% CI)	p-value
Australia	Male sex	1.03 (0.92–1.14)	0.01 (-0.05–0.08)	0.633
	Age<44 years	1.04 (0.91–1.19)	0.02 (-0.06–0.10)	0.575
	Unsatisfied with standard of living	1.32 (1.08–1.61)	0.18 (0.03–1.33)	**0.006**
Hong Kong	Male sex	1.24 (1.04–1.46)	0.10 (0.02–0.17)	**0.013**
	Age <44 years	0.87 (0.74–1.04)	-0.06 (-0.14–0.02)	0.126
Japan	Age <44 years	1.14 (1.07–1.22)	0.10 (0.06–0.16)	**<0.001**
	Unsatisfied with health	1.10 (1.02–1.18)	0.08 (0.02–0.14)	**0.014**
South Korea	Age <44 years	1.08 (1.00–1.16)	0.06 (<0.01–0.11)	**0.043**
	Unsatisfied with standard of living	1.17 (1.09–1.26)	0.13 (0.07–0.19)	**<0.001**
Taiwan	Age <44 years	1.16 (1.05–1.27)	0.09 (0.03–0.14)	**0.002**
	Unsatisfied with standard of living	1.17 (1.05–1.30)	0.10 (0.03–0.17)	**0.004**
Thailand	Age <44 years	1.29 (1.07–1.56)	0.07 (0.02–0.12)	**0.007**
	Unsatisfied with standard of living	1.67 (1.27–2.18)	0.23 (0.10–0.35)	**<0.001**
	Unsatisfied with health	1.65 (1.26–2.16)	0.19 (0.07–0.31)	**<0.001**

Table 3 presents the regression models for the predictors of lower trust in political leaders for each country. Models were calculated for all six countries. The general trend across all countries is that males, people aged under 44 years (the median age) and people unsatisfied with either their health or standard of living are more likely to have lower trust in political leaders. The majority of the statistically significant RRs are relatively low, in the magnitude 1.1–1.3, although in Thailand, people who are unsatisfied with their health and standard of living are almost 70% (RR1.7; 95%CI 1.3–2.2 and RR1.7; 95%CI 1.3–2.2 respectively) more likely to have lower trust in political leaders than people who are satisfied with their health and standard of living.

Table 4 presents the regression models for the predictors of lower trust in press/newspapers for each country. Only two models were calculated for lower trust in the newspapers, namely Japan and Thailand. In Japan, the predictors of lower trust in the press were being male, under 44 years and unsatisfied with health, all of which has RRs between 1.5–1.7. In Thailand, the single predictor of lower trust in newspapers was being unsatisfied with health (RR1.6; 95%CI 1.1–2.2). All predictors of lower trust in newspapers in these two countries suggest a 50–70% increase likelihood of lower trust for these groups.

**Table 4:** Multivariate regression models for lower trust (trust ‘not at all’ or ‘not much’) in Press/Newspapers in each country.

Country	Characteristic	Risk Ratio (95% CI)	Risk Difference (95% CI)	p-value
Japan	Male sex	1.50 (1.08–2.08)	0.04 (<0.01–0.08)	**0.016**
	Age <44 years	1.65 (1.20–2.28)	0.06 (0.02–0.10)	**0.002**
	Unsatisfied with Health	1.58 (1.11–2.23)	0.06 (0.01–0.12)	**0.010**
Thailand	Age <44 years	1.03 (0.84–1.27)	0.06 (-0.05–0.18)	0.750
	Unsatisfied with standard of living	1.26 (0.89–1.78)	0.14 (0.03–0.26)	0.201
	Unsatisfied with health	1.59 (1.14–2.23)	0.23 (0.20–0.25)	**0.007**

Table 5 presents the regression models for the predictors of lower trust in TV/media for each country. Four models were calculated for lower trust in TV, namely Hong Kong, Japan, South Korea and Thailand. Similar to the previous institutions, key predictors of lower trust in TV were being male, aged under 44 years and being unsatisfied with health and standard of living. However, people living without a partner were also more likely to have lower trust in TV in Japan and South Korea, albeit relatively low RRs (RR1.3; 95%CI 1.01–1.5 and RR1.2; 95%CI 1.0–1.4) suggesting a lower explanatory power than the other variables. People unsatisfied with their health were 60% more likely in Hong Kong (RR1.6; 95%CI 1.2–2.0) and twice as likely in Thailand (RR2.1; 95%CI 1.5–2.8) to have lower trust in TV than people satisfied with their health.

**Table 5:** Multivariate regression models for lower trust (trust ‘not at all’ or ‘not much’) in TV/Media in each country.

Country	Characteristic	Risk Ratio (95% CI)	Risk Difference (95% CI)	p-value
Hong Kong	Age<44 years	1.08 (0.90–1.30)	0.03 (-0.05–0.12)	0.405
	Unsatisfied with Health	1.59 (1.24–2.04)	0.23 (0.08–0.38)	**<0.001**
Japan	Male sex	1.27 (1.03–1.57)	0.04 (-0.04–0.11)	**0.024**
	Living without a partner	1.25 (1.01–1.54)	0.11 (-0.03–0.24)	**0.037**
South Korea	Age <44 years	1.14 (0.96–1.36)	0.05 (-0.01–0.11)	0.126
	Living without a partner	1.15 (0.97–1.37)	0.06 (-0.01–0.13)	**0.107**
Thailand	Unsatisfied with standard of living	1.64 (1.17–2.28)	0.12 (0.02–0.22)	**0.006**
	Unsatisfied with health	2.05 (1.52–2.75)	0.19 (0.09–0.29)	**<0.001**

Table 6 presents the regression models for the predictors of lower trust in the judicial/legal system for each country. Four models were calculated for lower trust in the legal system, namely Japan, South Korea, Taiwan and Thailand. The key predictors, similar to previous lower trust in other institutions, were people aged under 44 years and people unsatisfied with their health or standard of living. Most predictors had relatively low RRs, except for Thailand where people unsatisfied with their health or standard of living were over twice as likely to have lower trust in the legal system than people who were satisfied (RR2.2; 95%CI 1.4–3.6 and RR2.4; 95%CI 1.5–3.8 respectively).

**Table 6:** Multivariate regression models for lower trust (trust ‘not at all’ or ‘not much’) in Judicial/Legal System in each country.

Country	Characteristic	Risk Ratio (95% CI)	Risk Difference (95% CI)	p-value
Japan	Age <44 years	1.36 (1.11–1.67)	0.06 (-0.01–0.13)	**0.003**
	Unsatisfied with health	1.47 (1.24–1.91)	0.09 (0.03–0.16)	**<0.001**
South Korea	Male sex	1.13 (1.01–1.26)	0.07 (0.01–0.13)	**0.031**
	Age <44 years	1.16 (1.03–1.29)	0.08 (0.02–0.14)	**0.012**
Taiwan	Unsatisfied with standard of living	1.23 (1.04–1.46)	0.10 (0.01–0.18)	**0.019**
	Unsatisfied with health	1.11 (0.91–1.35)	-0.04 (-0.04–0.16)	0.316
	Living *with* a partner	1.15 (1.01–1.31)	<0.01 (<0.01–0.12)	**0.039**
Thailand	Age <44 years	1.19 (0.85–1.66)	0.14 (0.01–0.07)	**0.297**
	Unsatisfied with standard of living	2.36 (1.49–3.75)	0.17 (0.06–0.28)	**<0.001**
	Unsatisfied with health	2.23 (1.39–3.56)	0.15 (0.05–0.26)	**0.001**

Table 7 presents the regression models for the predictors of lower trust in Banks for each country. This question was not asked in the Japanese study, and all other countries had a calculated regression model. The key predictors of lower trust in banks were being male, under 44 years and unsatisfied with health or standard of living. The majority of the RRs were between 1.2–1.8, suggesting a low to moderate increase in lower trust for these groups, although in Thailand, people who were unsatisfied with their health were over three times more likely to have lower trust than people satisfied with their health (RR3.3; 95%CI 1.9–5.7).

**Table 7:** Multivariate regression models for lower trust (trust ‘not at all’ or ‘not much’) in Banks in each country.

Country	Characteristic	Risk Ratio (95% CI)	Risk Difference (95% CI)	p-value
Australia	Male sex	1.16 (1.03–1.31)	0.07 (0.01–0.13)	**0.014**
	Age <44 years	1.24 (1.08–1.42)	0.11 (0.03–0.13)	**0.002**
Hong Kong	Male sex	1.26 (0.95–1.67)	0.06 (-0.01–0.13)	0.102
	Age ≥44 years	1.47 (1.12–1.95)	0.09 (0.03–0.16)	**0.006**
South Korea	Male sex	1.18 (1.01–1.37)	0.08 (0.01–0.13)	**0.035**
	Age <44 years	1.32 (1.13–1.55)	0.11 (0.05–0.17)	**<0.001**
Taiwan	Male sex	1.20 (1.00–1.45)	0.03 (-0.02–0.08)	**0.054**
	Age <44 years	1.45 (2.20–1.76)	0.09 (0.04–0.14)	**<0.001**
	Unsatisfied with standard of living	1.58 (1.29–5.94)	0.13 (0.06–0.21)	**<0.001**
Thailand	Age <44 years	1.33 (0.89–1.98)	0.02(-0.01–0.05)	0.171
	Unsatisfied with standard of living	1.85 (1.06–3.22)	0.08 (-0.03–0.17)	**0.029**
	Unsatisfied with health	3.31 (1.93–5.68)	0.18 (0.08–0.29)	**<0.001**

Table 8 presents the regression models for the predictors of lower trust in Religious Organisations for each country. This question was not asked in the Japanese study, and all other countries except Hong Kong had a calculated regression model. In Australia, South Korea and Taiwan, the only predictors of lower trust in Religious Organisations was being male and under 44 years. The RRs in these models were 1.2–1.5, suggesting fairly low explanatory power. The model in Thailand had a single variable, people unsatisfied with their standard of living, who we over twice as likely to have lower trust in Religious Organisations than people who were satisfied with their standard of living (RR2.3; 95%CI 1.3–4.0).

**Table 8:** Multivariate regression models for lower trust (trust ‘not at all’ or ‘not much’) in Religious Organisations in each country.

Country	Characteristic	Risk Ratio (95% CI)	Risk Difference (95% CI)	p-value
Australia	Male sex	1.35 (1.15–1.59)	0.11 (0.04–0.17)	**<0.001**
	Age <44 years	1.45 (1.22–1.73)	0.14 (0.06–0.22)	**<0.001**
South Korea	Male sex	1.24 (1.11–1.40)	0.10 (0.04–0.16)	**<0.001**
	Age <44 years	1.20 (1.07–1.35)	0.11 (0.05–0.17)	**0.002**
Taiwan	Male sex	1.26 (1.05–1.51)	0.06 (0.01–0.11)	**0.012**
	Age <44 years	1.54 (1.28–1.86)	0.12 (0.07–0.17)	**<0.001**
	Unsatisfied with standard of living	1.15 (0.93–1.44)	0.04 (-0.04–0.11)	0.204
Thailand	Age <44 years	1.50 (0.90–2.51)	0.02 (-0.01–0.05)	0.119
	Unsatisfied with standard of living	2.26 (1.27–4.03)	0.07 (<0.01–0.14)	**0.006**
	Living without a partner	1.35 (0.86–2.12)	0.02 (-0.02–0.05)	0.190

## Discussion

Citizens in democratic societies are expected to engage in trust relations with governments, banks, the media and the legal system, as trust is integral to maintaining the smooth functioning of society [41,57]. In order to create and maintain trustworthiness, politicians are called upon to follow through with election promises, bankers to run their institutions in the best interests of their customers and lawyers/judges to maintain social order through the courts. In this way, trust is the basis for a well-organised democratic society [58].

The representatives of major institutions such as politics, banks and the judicial system are acutely aware of the importance of gaining and maintaining citizens’ trust in their institutions [42], although trust in politicians and national governments is not always high [28,29,59]. Levi [12] argued that governments and other institutions need to be regarded as legitimate and ‘doing their job’ in the eyes of citizens, since legitimacy is a key requirement for trustworthiness. This rather positive view of the transparency suggests a central place for citizens and a need for institutions to ‘model’ trustworthiness and trust-building/maintenance strategies. Transparency in government has been described as a ‘basic human right’ [29]. However, Hardin argued that most citizens lack the information required to decide whether a government is trustworthy [38]. Furthermore, Peel [60] argues that discussions about Australian political culture ‘have tended to assume that distrust of politicians and governments stem from declining civic awareness or a lack of civic education. The problem, in other words, lies within the citizens’ (p. 315), thereby disregarding the need for institutions to actively engage in trust building. However, research in South Korea found that increasing levels of transparency actually lead to reduced perceptions of competency of government [29]. Trust is viewed by a some philosophers and sociologists as a “social practice and process because it involves the responsibility of both parties, commitment to the relationship, and the possibility of social change” [61] (p.125). Our research suggests that the social practice of trust may differ according to social, cultural, political and economic systems, necessitating greater interpretation of our data from researchers within each of the participating countries.

The central hypothesis within this paper focused on the Confucianist societies having lower institutional trust than non-Confucianist societies, based on Fukuyama’s theory [41] while also acknowledging that there lies a spectrum of trust that affords not only high- and low-trust societies, but also what we regard as medium-trust societies. In order to corroborate this theory, we would have expected Taiwan and Hong Kong to have lowest levels of institutional trust, followed by Thailand and South Korea with ‘medium levels’ of institutional trust and then Australia and Japan with the highest levels of trust. However, the picture that our data painted did not conform to this pattern. Data in Tables 1 and 2 show that, based on overall levels of trust across all institutions, Thailand can generally be seen as a ‘higher trust society, followed by Hong Kong and Japan as ‘medium trust societies’ and finally Australia, South Korea and Taiwan as ‘lower trust societies’. In making the judgements about the ‘level’ of trust in each country from our data, we have made relative rather than absolute determinations. For example, we have classified Australia, South Korea and Taiwan as ‘lower trust’, since the levels were lower than the other countries in the dataset, although there was still between 43–55% of respondents in these countries having higher trust in institutions.

There were differences in the countries in terms of trust in different organisations, which demonstrates the problematic nature of classifying countries as high or low trusting. For example, we classified Japan as a ‘medium trust society’, although 25% of Japanese respondents had higher trust in political leaders (lower trust) whereas 87% had higher trust in newspapers (higher trust). Australia was classified as a ‘low trust society’, although 24% had higher trust in the TV, whereas 62% had higher trust in religious organisations. Hong Kong was classified as a ‘medium trust society’, although there was higher trust in TV (62%) but much lower trust in political leaders (25%). Thailand had fairly stable levels of higher trust across all institutions and Taiwan and Hong Kong had fairly stable levels of medium trust across all institutions.

The second main aim of the paper was to understand the key predictors of trust in different countries, in order to identify population groups with lower or higher trust, and the similarities and differences between countries. In Australia, those who reported being unsatisfied with their standard of living reported the lowest trust in political leaders, and those under the median age of 44 years reported the lowest trust in banks. Men and people under 44 years of age reported the lowest trust in religious organisations. In Hong Kong, males reported significantly low levels of trust in their political leaders, and those who reported being unsatisfied with their health reported significantly low levels of trust in their TV/media. In Japan, people under the age of 44 reported significantly lower trust in their political leaders and judicial/legal system. Males reported lower trust in their press/newspapers and TV/media. People with a lack of satisfaction with their health also reported poor trust in press/newspapers and the judicial/legal system. In South Korea, respondents under the age of 44 reported lower levels of trust in their political leaders, judicial system, banks and religious organisations. Men also reported lower levels of trust in their judicial/legal system, banks and religious organisations. Those who reported being unsatisfied with their standard of living also reported low trust in their political leaders. In Taiwan, those who reported being unsatisfied with their standard living reported lower levels of trust in their political leaders, judicial/legal system and banks. Respondents under 44 years reported lower levels of trust in political leaders, banks and religious organisations. Males also reported lower trust in religious organisations. In Thailand, those who reported being unsatisfied with their health reported significantly lower trust in their political leaders, press/newspapers, judicial/legal system and banks. Those unsatisfied with their standard of living reported very lower trust in their political leaders, judicial/legal system, banks and religious organisations. Respondents under 44 years of age also reported lower levels of trust in their political leaders.

In the broad literature on institutional trust, researchers generally report higher levels of trust by people with higher incomes and higher education levels [13,27]. For example, Freitag [62] found that higher education and income lead to more social trust (trust in others), suggesting that education makes people more ‘open minded’ and thus able to make reflexive choices about trust. Likewise, it has been found that people with higher levels of education display significantly more trust in government and less trust in tabloid newspapers compared with people with fewer years of education [63]. In addition, the ‘rational actor’ model of trust assumes that people can only trust what they know, therefore, more knowledge potentially increases trust [38] up to the point where trust is no longer required (complete knowledge). People with higher social trust have also been found to have higher political trust and vice versa [64,65], suggesting that people on lower incomes and with less education are likely to have lower trust in politics, political parties and potentially other institutions of power and hierarchy in society. Indeed, this links with Putnam’s ideas [66] on trust and social capital, whereby he argues that the ‘have nots’ in all societies are less trusting than the ‘haves’, since the ‘have nots’ are treated with less respect. Whilst education was a variable in our analysis, it did not come out as a predictor of institutional trust.

Within our analysis, the predictors of ‘low satisfaction with health’ and ‘low satisfaction with standard of living’ were key predictors of lower trust across most countries and institutions. Both of these variables suggest forms of disadvantage, and were also predictors of low trust in the media across 29 Asian countries [27]. Peel argues that for disadvantaged populations, distrust is a rational, critical response to their actual experiences of distrustful and even destructive governance [60], arguing that government promises for disadvantaged communities are often ‘empty’ and that ‘nothing ever gets done’, reflecting research undertaken in the UK [67] and Australia [59]. People’s perceptions of whether or not an institution such as the government, banks or the legal system is performing well (and thus worthy of trust) depends largely on whether they provide services that are useful to that particular individual [68]. However, public perceptions of most institutions depend to some extent on media reports of service provision [69], making clear the role of the media in co-constructing public perceptions of the trustworthiness of institutions. Baudrillard termed this ‘simulacra’ [70], which suggests a representation rather than ‘reality’ per se. The importance of the media in influencing public trust in institutions is highlighted by evidence of a relationship between the volume of media reporting and people’s perception of risk that is unrelated to the generalised level of trust in the media [71,72], a convergence of the values of readers of elite press with media presentations over time [73]. It has been argued that the media does not provide an adequate conduit for unbiased reporting of stories [74], increasing plurality in the views presented and greater critique of science, all of which have the potential to increase uncertainty and question trustworthiness of the media and the institutions they report on [75].

In conclusion, within this paper we analysed data on institutional trust in six Asia-Pacific countries, specifically focusing on trust in political leaders, banks, TV/media, press/newspapers, judicial/legal system and religious organisations. We interpreted our data using Fukuyama’s theory of ‘high/low trust’ societies, taking into account the possibility of identifying what we have referred to as ‘medium-trust’ societies. The levels of institutional trust in each society did not conform to our hypothesis, with Thailand exhibiting the highest trust (predicted to be medium level), Hong Kong and Japan exhibiting medium trust (predicted to be low and high respectively) and Australia and South Korea exhibiting low trust (predicted to be high and medium respectively). Taiwan was the only country where the actual and predicted trust was the same, namely low trust. In terms of the different institutions, there was generally highest trust in religious organisations, banks and the judicial/legal system (between 67–70%), followed by press/newspapers and TV/media (59% and 58% respectively) and lowest trust in political leaders (43%). The key predictors of lower trust in institutions across all countries were males, people under 44 years and people unsatisfied with the health and standard of living. Given the fact that these predictors crossed national boundaries and institutional types, further research and policy should focus specifically on improving trust within these groups in order that they can be empowered to play a more central role in democratic vitality.

## References

[1] Mollering G (2001) The nature of trust: From Georg Simmel to a theory of expectation, interpretation and suspension. Sociology 35: 403–420. 10.1177/s0038038501000190

[2] Simmel G (1990) The Philosophy of Money. London: Routledge.

[3] Luhmann N (1979) Trust and Power. New York: Wiley.

[4] Meyer S, Ward PR, Coveney J, Rogers W (2008) Trust in the health system: an analysis and extension of the social theories of Giddens and Luhmann. Health Sociology Review 17: 177–186. 10.5172/hesr.451.17.2.177

[5] Shepard BH, Sherman DM (1998) The Grammars of Trust and General Implications. Academy of Management Review 23: 422–438.

[6] Govier T (1998) Dilemmas of Trust. Montreal: McGill-Queen's University Press.

[7] Sabel CF (1993: 1133) Studied trust: Building new forms of cooperation in a volatile economy. Human Relations 45: 1133–1170.

[8] Giddens A (1994) Risk, Trust, Reflexivity In: Beck U, Giddens A, Lash S, editors. Reflexive Modernization. Cambridge: Polity Press.

[9] Sztompka P (1999: 6) Trust: A Sociological Theory. Cambridge: Cambridge University Press.

[10] Warren M (2004) Democratic theory and trust In: Warren M, editor. Democracy and Trust. Cambridge: Cambridge University Press.

[11] Kampen J, De Walle S, Bouckaert G (2006) Assessing the relation between satisfaction with public service delivery and trust in Government. The impact of the predisposition of citizens toward Government on evalutations of its performance. Public Performance & Management Review 29: 387–404.

[12] Levi M (1998) A State of Trust In: Braithwaite V, Levi M, editors. Trust tand Governance. New York State: Russell Sage Foundation.

[13] Tsfati Y, Ariely G (2014) Individual and contextual correlates of trust in media across 44 countries. Communication Research 41: 760–782. 10.1177/0093650213485972

[14] Tsfati Y, Cohen J (2005) Democratic consequences of hostile media perceptions: the case of Gaza settlers. Harvard International Journal of Press/Politics 10: 28–51. 10.1177/1081180x05280776

[15] Fukuyama F (2010) Transitions to the Rule of Law. Journal of Democracy 21: 33–44. 10.1353/jod.0.0145

[16] Ward P, Mamerow L, Meyer S (2013) Identifying vulnerable populations using a Social Determinants of Health framework: analysis of national survey data across six Asia-Pacific countries. PLOS ONE 8 e83000 10.1371/journal.pone.0083000 24349417 PMC3857316

[17] Meyer S, Luong T, Mamerow L, Ward P (2013) Inequities in access to healthcare: analysis of national survey data across six Asia-Pacific countries. BMC Health Services Research 13: 238 10.1186/1472-6963-13-238 23816181 PMC3734194

[18] Ward P, Mamerow L, Meyer S (2014) Interpersonal trust across six Asia-Pacific countries: testing and extending the 'high trust society' and 'low trust society' theory. PLOS ONE 9: e95555 10.1371/journal.pone.0095555 24760052 PMC3997396

[19] Kehr F, Kowatsch T, Wentzel D, Fleisch E (2015) Blissfully ignorant: the effects of general privacy concerns, general institutional trust, and affect in the privacy calculus. Information Systems Journal 25: 607–635. 10.1111/isj.12062

[20] Xiao C, McCright A (2015) Gender differences in environmental concern: Revisiting the institutional trust hypothesis in the USA. Environment and Behavior 47: 17–37.

[21] Park S (2012) Toward the trusted public organization: Untangling the leadership, motivation and trust relationship in U.S. Federal Agencies. The American Review of Public Administration 42: 562–590. 10.1177/0275074011410417

[22] Hmielowski J, Feldman L, Myers T, Leiserowitz A, Maibach E (2014) An attack on science? Media use, trust in scientists, and perceptions of global warming. Public Understanding of Science 23: 866–883. 10.1177/0963662513480091 23825287

[23] Cole L, Cohn E (2016) Institutional Trust Across Cultures: Its Definitions, Conceptualizations, and Antecedents Across Eastern and Western European Nations In: Shockley E, Neal T, PytlikZillig L, Bornstein B, editors. Interdisciplinary Perspectives on Trust: Springer International Publishing pp. 157–176.

[24] Hooghe M, Verhaegen S (2015) The effect of political trust and trust in European citizens on European identity. European Political Science Review: 1–21. 10.1017/s1755773915000314

[25] Muñoz J, Torcal M, Bonet E (2011) Institutional trust and multilevel government in the European Union: Congruence or compensation? European Union Politics 12: 551–574. 10.1177/1465116511419250

[26] Hudson J (2006) Institutional trust and subjective wellbeing across the EU. Kyklos 59: 43–62. 10.1111/j.1467-6435.2006.00319.x

[27] Tokuda Y, Fujii S, Jimba M, Inoguchi T (2009) The relationship between trust in mass media and the healthcare system and individual health: evidence from the AsiaBarometer Survey. BMC Medicine 7: 1–10. 19161600 10.1186/1741-7015-7-4 PMC2655302

[28] Wang C-H (In Press) Government Performance, Corruption, and Political Trust in East Asia. Social Science Quarterly.

[29] Grimmelikhuijsen S, Porumbescu G, Hong B, Im T (2013) The Effect of Transparency on Trust in Government: A Cross-National Comparative Experiment. Public Administration Review 73: 575–586. 10.1111/puar.12047

[30] Bureekul T, Thananithichot S (2013) Trust and Social Cohesion, the Key to Reconcile Thailand Future. International Journal of Social Quality 2.

[31] Hu K, Chan RKH (2013) Social Capital and Civic Engagement in Urban China. International Journal of Social Quality 2.

[32] Giddens A (1990) The Consequences of Modernity. Cambridge: Polity Press.

[33] Gambetta D (1988) Trust: Making and Breaking Co-operative Relations. Oxford: Basil Blackwell.

[34] Brown PR, Meyer SB (2015) Dependency, trust and choice? Examining agency and ‘forced options’ within secondary-healthcare contexts. Current Sociology 10.1177/0011392115590091

[35] Barbalet J (2009) A characterization of trust, and its consequences. Theory and Society 38: 367–382. 10.1007/s11186-009-9087-3

[36] Luhmann N (2000) Familiarity, Confidence, Trust: Problems and Alternatives In: Gambetta D, editor. Trust: Making and Breaking Cooperative Relations. Oxford: Blackwell.

[37] Luhmann N (1988) Trust: Making and Breaking Cooperative Relations In: Gambetta D, editor. Familiarity, Confidence, Trust: Problems and Alternatives. New York: Basil Blackwell pp. 94–107.

[38] Hardin R (2006) Trust. Cambridge: Polity Press.

[39] Mishler W, Rose R (2001) What are the origins of political trust? Testing institutional and cultural theories in post-Communist societies. Comparative Political Studies 34 10.1177/0010414001034001002

[40] Hardin R (1998) Trust in Government In: Braithwaite V, Levi M, editors. Trust and Governance. New York: Russell Sage Foundation.

[41] Fukuyama F (1996) Trust: the Social Virtues and Creation of Prosperity. London: Free Press.

[42] Braithwaite V (1998) Communal and exchnage trust norms: their value base and relevance to institutional trust In: Braithwaite V, Levi M, editors. Trust and Governance. New York: Russell Sage Foundation.

[43] Khodyakov D (2007) Trust as a process: A three-dimensional approach. Sociology 41: 115–132. 10.1177/0038038507072285

[44] Brown PR (2009) The phenomenology of trust: a Schutzian analysis of the social construction of knowledge by gynae-oncology patients. Health, Risk & Society 11: 391–407. 10.1080/13698570903180455

[45] Kuwabara K, Willer R, Macy M, Mashima R, Terai S, et al (2007) Culture, Identity, and Structure in Social Exchange: A Web-based Trust Experiment in the United States and Japan. Social Psychology Quarterly 70: 461–479. 10.1177/019027250707000412

[46] Wang F, Yamagishi T (2005) Group-based trust and gender differences in China. Asian Journal of Social Psychology 8: 199–210. 10.1111/j.1467-839x.2005.00167.x

[47] Yamagishi T, Yamagishi M (1994) Trust and commitment in the United States and Japan. Motivation and Emotion 18: 129–166. 10.1007/bf02249397

[48] Chen F-l, Shi S-J (2013) Social Exclusion Experiences of Atypical Workers: A Case Study of Taipei. International Journal of Social Quality 2.

[49] Vajirakachorn S (2013) Social Inclusion in Southern Border Provinces of Thailand International Journal of Social Quality 2.

[50] Ward P, Meyer S, Verity F, Gill T, Luong T (2011) Complex problems require complex solutions: the utility of social quality theory for addressing the Social Determinants of Health. BMC Public Health 11: 630 10.1186/1471-2458-11-630 21819576 PMC3167771

[51] World Values Survey Association (2005/2006) World Values Survey. http://www.worldvaluessurvey.org/.

[52] National Opinion Research Center General Social Survey. Chicago: NORC www3.norc.org/gss+website/.

[53] Bowling A, editor (2009) Research Methods in Health. 3rd ed. New York: Open University Press.

[54] Hosmer DW, Lemeshow S (2000) Applied Logistic Regression. New Jersey: John Wiley & Sons.

[55] Nagelkerke NJD (1991) A note on a general definition of the coefficient of determination. Biometrika 78: 691–692. 10.1093/biomet/78.3.691

[56] Field A (2009) Discovering Statistics using SPSS (3rd edition). London: Sage.

[57] Van der Schee E, Baum B, Calnan M, Schnee M, Groenowegen P (2007) Public trust in health care: a comparison of Germany, The Netherlands, and England and Wales. Health Policy 81: 56–67. 10.1016/j.healthpol.2006.04.004 16777257

[58] Misztal BA (1996) Trust in Modern Societies. Cambridge: Polity Press.

[59] Meyer S, Mamerow L, Taylor A, Henderson J, Ward P, et al (2012) Demographic indicators of trust in Federal, State and local government: Implications for Australian health policy makers. Australian Health Review 37: 11–18. 10.1071/AH11073 23116546

[60] Peel M (1998) Trusting disadvantaged citizens In: Braithwaite V, Levi M, editors. Trust and Governance. New York: Russell Sage Foundation.

[61] Khodyakov D (2007) Trust as a Process: A Three-Dimensional Approach. Sociology 4: 115–132.

[62] Freitag M (2003) Social capital in (dis)similar democracies: the developemnt of generalised trust in Japan and Switzerland. Comparative Political Studies 36: 936–966. 10.1177/0010414003256116

[63] Frewer L, Howard C, Hedderley D, Shepherd R (1998) Methodological approaches to assessing risk perceptions associated with food-related hazards. Risk Analysis 18: 95–102. 10.1111/j.1539-6924.1998.tb00919.x 9523447

[64] Brewer P, Gross K, Aday S, Willnat L (2004) International trust and public opinion about World Affairs. American Journal of Political Science 48: 93–109. 10.1111/j.0092-5853.2004.00058.x

[65] Sønderskov K, Dinesen P (2015) Trusting the State, trusting each other? The effect of institutional trust on social trust. Political Behavior: 1–24.

[66] Putnam R (2000) Bowling Alone: The Collapse and Revival of American Community. New York: Simon & Schuster.

[67] Ward P, Coates A (2006) "We shed tears but there is no one there to wipe them up for us": narratives of (mis)trust in a materially deprived community. Health: An interdisciplinary journal for the social study of health, illness and medicine 10: 283–302. 10.1177/1363459306064481 16775016

[68] Job J (2005) How is trust in the government created? It begins at home, but ends in the parliament. Australian Review of Public Affairs 6: 1–23.

[69] Ward P, Henderson J, Coveney J, Meyer S (2012) How do Australian consumers negotiate and respond to information in the media about food and nutrition?: the importance of risk, trust and uncertainty. Journal of Sociology 48: 21–39.

[70] Baudrillard J (1994) Simulacra and Simulation. Michigan: University of Michigan Press.

[71] Frewer L, Scholderer J, Bredahl L (2003) Communicating about the risks and benefits of genetically modified foods: the mediating role of trust. Risk Analysis 23: 1117–1133. 10.1111/j.0272-4332.2003.00385.x 14641888

[72] Frewer L, Miles S, Marsh R (2002) The media and genetically modified foods: evidence in support of social amplification of risk. Risk Analysis 22: 701–711. 10.1111/0272-4332.00062 12224744

[73] Bauer M (2015) Atoms, Bytes and Genes. Public Resistance and Techno-Scientific Responses. London: Routledge.

[74] Henderson J, Wilson A, Meyer S, Coveney J, Calnan M, et al (2014) The role of the media in construction and presentation of food risks. Health, Risk & Society 16: 615–630. 10.1080/13698575.2014.966806

[75] Schafer M (2009) From public understanding to public engagement: An empirical assessment of changes in science reporting. Science Communication 30: 475–505.

